# Reliability of a method to assess corticomotor excitability of lower limb muscles using a normalized EMG motor thresholding procedure

**DOI:** 10.1038/s41598-024-51622-6

**Published:** 2024-01-24

**Authors:** Yo Shih, Christopher M. Powers, Beth E. Fisher

**Affiliations:** 1 Department of Rehabilitation Sciences, University of Oklahoma Health Sciences Center, Oklahoma City, OK USA; 2 Division of Biokinesiology and Physical Therapy, University of Southern California, Los Angeles, CA USA

**Keywords:** Translational research, Outcomes research, Motor control, Movement disorders

## Abstract

Given the importance of determining intervention-induced neuroplastic changes with lower extremity functional tasks, a reliable transcranial magnetic stimulation (TMS) methodology for proximal lower extremity muscles is needed. A pre-set fixed voltage value is typically used as the criterion for identifying a motor evoked potential (MEP) during the motor thresholding procedure. However, the fixed voltage value becomes problematic when the procedure is applied to proximal lower extremity muscles where active contractions are required. We sought to establish the reliability of a method measuring corticomotor excitability of gluteus maximus and vastus lateralis using normalized electromyography (EMG) as the criterion for identifying MEPs during the motor thresholding procedure. The active motor threshold for each muscle was determined using the lowest stimulator intensity required to elicit 5 MEPs that exceeded 20% maximal voluntary isometric contraction from 10 stimulations. TMS data were obtained from 10 participants on 2 separate days and compared using random-effect intra-class correlation coefficients (ICCs). Slopes from two input–output curve fitting methods as well as the maximum MEP of gluteus maximus and vastus lateralis were found to exhibit good to excellent reliability (ICCs ranging from 0.75 to 0.99). The described TMS method using EMG-normalized criteria for motor thresholding produced reliable results utilizing a relatively low number of TMS pulses.

## Introduction

Transcranial magnetic stimulation (TMS) is a research method that can be used to probe the excitability of the corticospinal descending pathways of the central nervous system^1,2^. TMS measures have been shown to be related to motor behaviors such as reaction time^3^, gait function^4^, as well as skill training induced-neuroplasticity^5^. TMS provides neurophysiological information that may provide insight into how the central nervous system contributes to the modulation of movement behavior. There is a growing interest in the TMS assessment of lower extremity muscles such as the gluteus maximus and vastus lateralis to understand how excitability of the corticospinal tract may contribute to various lower extremity injuries^6–10^.

Although TMS protocols for upper extremity muscles have been well-established, there are inherent challenges associated with the attainment of reliable TMS measures of proximal lower extremity muscles^11^. Specifically, the cortical representational areas of proximal lower extremity muscles are small and lie within the medial longitudinal fissure thereby requiring use of a double cone coil^12^. In addition, an active contraction is necessary for measuring corticomotor excitability of proximal lower extremity muscles^12–14^. Muscle activation during the TMS procedure stabilizes cortical and spinal excitability and has been shown to elicit larger motor evoked potentials (MEPs) compared to MEPs at rest elicited by the same intensity^15,16^. However, the MEPs can be hidden and therefore unmeasurable within the EMG signal during active contractions. Given that the active state of a muscle is important in eliciting MEPs of proximal lower extremity muscles, standardizing the level of contraction during the motor thresholding procedure is important to facilitate the comparison of TMS responses between muscles and/or individuals.

The conventional method used for motor thresholding of upper extremity muscles is to determine the smallest stimulation intensity needed to elicit 5 out of 10 MEPs that are larger than a fixed voltage value (i.e. 100 or 200 uV). This method poses a challenge for the assessment of proximal lower extremity muscles as there are factors unrelated to muscle contraction that can influence the electromyographic (EMG) signal. In particular, the gluteus maximus has a greater amount of subcutaneous fat overlying the muscle compared to other lower extremity muscles such as the vastus lateralis. Subcutaneous fat is known to act as a low-pass filter thus attenuating EMG signals^17^.

The process of normalization is recommended to control for factors (ie. subcutaneous fat) that can interfere with comparison of EMG signals between muscles or among individuals^18^. The most common method used in kinesiologic EMG research is to express EMG signals as a percentage of a standardized level of activation (ie. maximum voluntary isometric contraction)^18^. Given the need to standardize the level of muscle contraction during the motor thresholding procedure, and to provide a comparable criterion for establishing MEPs, a TMS method that utilizes normalized EMG amplitudes would appear to have advantages over conventional methods that use fixed voltage values.

The purpose of the current study was to establish the reliability of a method for measuring corticomotor excitability of the gluteus maximus and vastus lateralis using normalized EMG intensity as the criterion for identifying MEPs during the motor thresholding procedure. We hypothesized that this method would result in acceptable between day reliability of various TMS outcome measures, thus facilitating comparison of data between muscles and/or individuals. Establishing a reliable TMS methodology for proximal lower extremity muscles is an important step in being able to measure intervention-induced neuroplastic changes with lower extremity functional tasks.

## Methods

### Participants

Ten healthy, active participants between the ages of 21 and 36 were recruited for this study (5 females, 5 males). Participants were excluded if they reported a previous history of lower extremity pathology or trauma, or lower extremity pain during sport or activities of daily living. Additional exclusion criteria included any affirmative answer on the TMS safety questionnaire indicating if they had metal, electrical, magnetic implants; a personal or family history of epilepsy; or the possibility of being pregnant^19^. Participants were required to attend 2 test sessions on 2 separate days within a week. All experimental protocols were approved by the Institutional Review Board of the Health Sciences Campus at the University of Southern California. Written informed consent was obtained from all participants included in the study. All methods were conducted in accordance with the relevant guidelines and regulations. Participants were asked to keep their daily routine consistent on both testing days (including caffeine intake).

### Maximal voluntary isometric contraction (MVIC) trials

**Figure 1:**
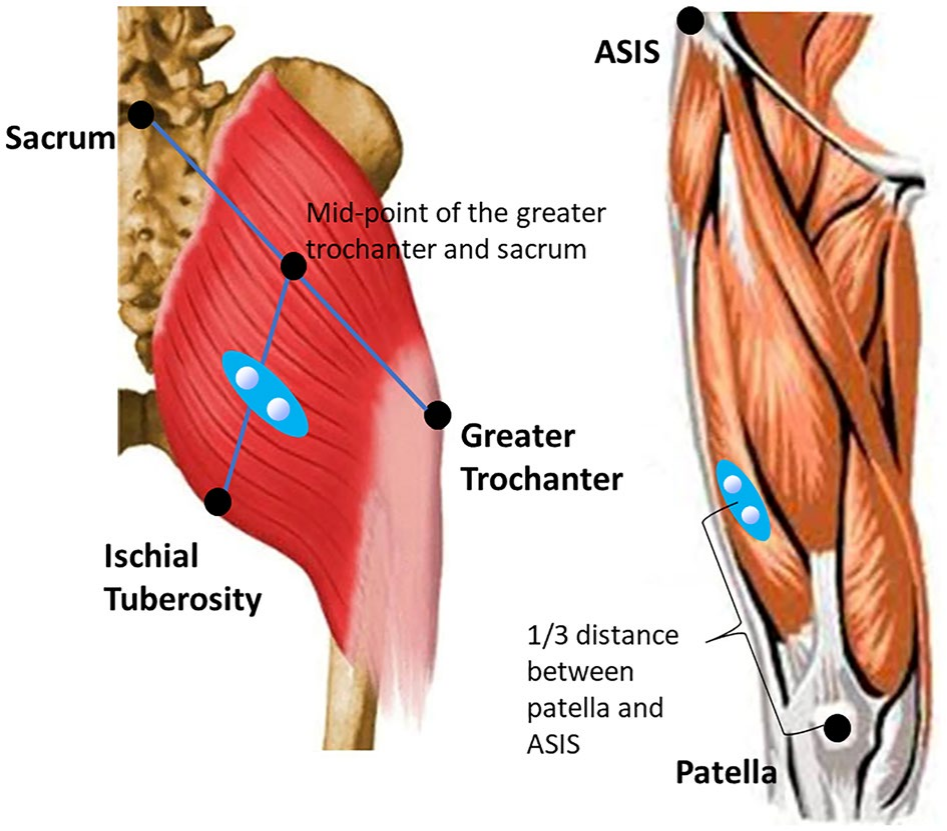
Placement of EMG electrodes for gluteus maximus and vastus lateralis.

Before initiating the TMS assessment, EMG signals of vastus lateralis and gluteus maximus were collected during a MVIC for the purposes of (1) providing the target contraction level during the TMS procedure, and (2) establishing the criteria for the identification of a MEP during motor thresholding. The skin over the vastus lateralis and gluteus maximus were shaved and prepared with alcohol to decrease skin impedance. Surface EMG electrodes (bipolar Ag/AgCl with 22mm inter-electrode distance) were placed over the muscle belly of vastus lateralis and gluteus maximus (Fig. 1). The electrode for vastus lateralis was positioned 1/3 of the distance between the patella and the anterior superior iliac spine^20^. For gluteus maximus, the electrode position was between the ischial tuberosity and the mid-point of a line connecting the the greater trochanter and the sacrum^20^. To allow for consistent electrode placement between testing days, electrode positions were marked with a permanent marker. The EMG signal was sampled at 5000 Hz and amplified using a gain of 2000.

Following electrode placement, MVIC trials of vastus lateralis and gluteus maximus were performed. For the MVIC trials of vastus lateralis, participants were seated with the hip and knee in 90° and 60° of flexion, respectively. A non-stretchable belt was placed at the distal end of the tibia to provide resistance. Participants were instructed to extend the knee against the belt with maximal effort and hold for 5 s. For the MVIC trials of gluteus maximus, participants were positioned prone with the hips at the edge of a treatment table. The tested leg was positioned in 90° of flexion, 45° of hip abduction, and end range of hip external rotation. Participants were instructed to push simultaneously into hip extension, abduction, external rotation against manual resistance provided by the examiner for 5 s^21^. Verbal encouragement was provided during the MVIC trials to facilitate a maximum effort. Two MVIC trials were performed for each muscle. The largest averaged 0.2 s root mean square value of the EMG signal from the two trials was used as the MVIC value for the TMS procedures described below.

### Settings

A single-pulse magnetic stimulator (MagStim 200^2^, The Magstim Company Ltd, Whitland, UK) with a 110 mm double cone coil was used for TMS assessments. A neuronavigation system (Brainsight, Rogue Research Inc, Montreal, Canada) was used as a guidance for accurate coil position during TMS assessments. Anatomical landmarks on the participants’ head including left and right ear, nasion and tip of nose were co-register on a 3-D reconstruction of a template magnetic resonance image of the brain. Clusters of reflective markers were attached on the coil and the participant’s forehead for tracking the relative position and orientation between the coil and the brain. All TMS stimulations were delivered at the primary motor cortex corresponding to the dominant leg (i.e. preferred kicking leg). Throughout the experiment, the inter-pulse interval was 5 s. The coil orientation was anterior–posterior.

### Positioning and instructions

Stimulations were delivered in the supine position during an active contraction of the gluteus maximus and vastus lateralis. For the gluteus maximus contraction, participants were instructed to “squeeze their buttocks”. For the vastus lateralis contraction, participants were instructed to “tighten their thigh muscle”. During the active contractions, real-time visual feedback reflecting the root mean square averaged amplitude of the EMG signal was provided. Participants were instructed to match the root mean square signal to the target contraction level set at 20% MVIC. Twenty percent MVIC was chosen as the target value. This value was chosen based on a previous study that reported better reliability of MEPs obtained from an upper extremity muscle during low level contractions (20–25% MVIC) compared to high level contractions (75–100% MVIC)^22^.

### Hot spotting

Prior to active motor thresholding, the optimal coil position on the scalp (i.e. “hotspot”) for the muscle of interest was identified by systematically delivering 5 stimulations to 25 locations within a 4 cm × 4 cm area lateral to the vertex of the contralateral side of the testing leg (as delineated in Brainsight). The hotspots of gluteus maximus and vastus lateralis were located separately and identified as the position on the scalp that elicited the largest and most consistent MEPs. The position of each hotspot was recorded in Brainsight so that stimulations could be applied to the same location for the second testing session.

### Motor thresholding

**Figure 2:**
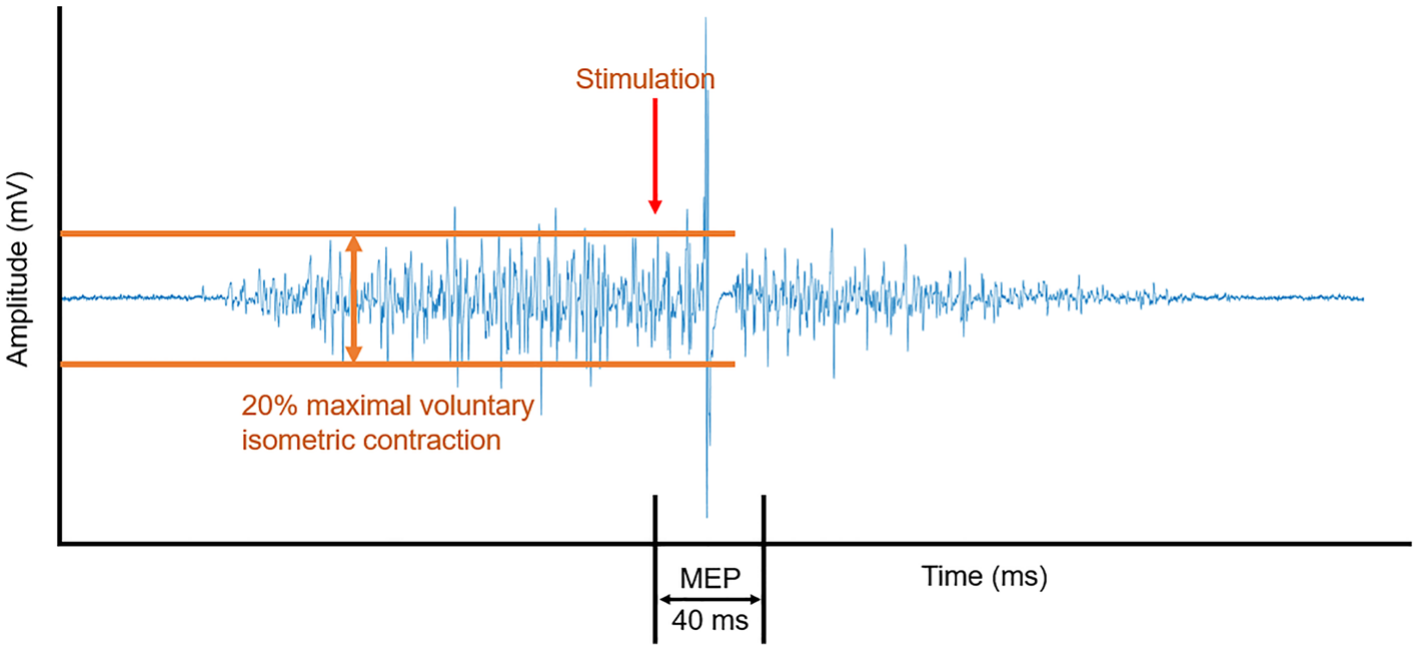
A demonstration of a MEP during a 20% MVIC.

Following the determination of the hotspot location, active motor thresholding was performed. The active motor threshold of gluteus maximus and vastus lateralis was determined as the smallest stimulation intensity that elicited 5 MEPs out of 10 stimulations. A MEP was identified as the TMS-induced EMG activation that was larger than background activation (> 20% MVIC) within 40 ms of the stimulation^1^ (Fig. 2).

### The input–output curve procedure

**Figure 3:**
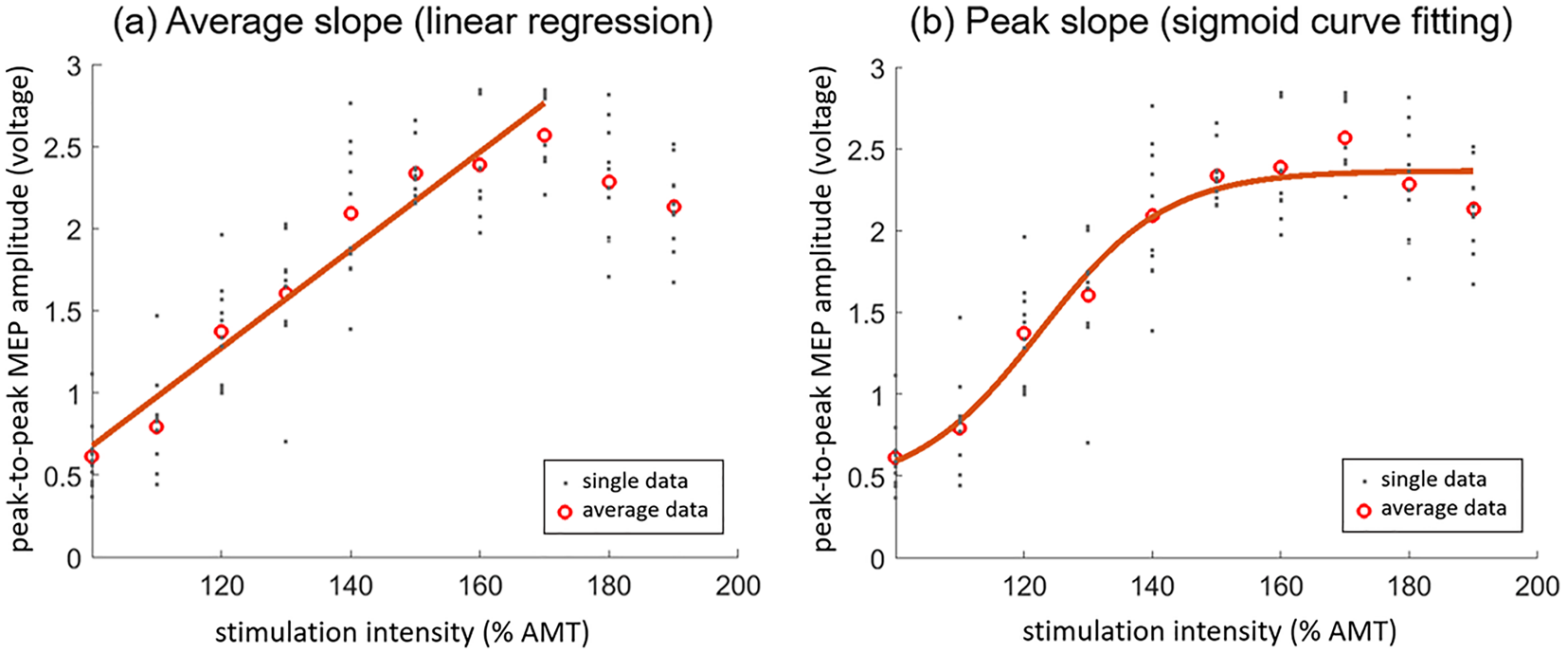
The input–output curve of one participant. The outcome of the input–output curve, the slope, was calculated from (**a**) linear regression fitting (the average slope), and (**b**) sigmoid curve fitting (the peak slope).

Following motor thresholding, the input–output curve procedure was performed for each muscle. Stimulations were performed over the hotspot at intensities ranging from 100 to 200% of active motor threshold in 10% increments. Ten stimulations were delivered at each of the stimulator intensities (Fig. 3).

### Data analysis

The average of the peak-to-peak amplitude of the 10 MEPs obtained at each stimulator intensity was calculated. An input–output curve for gluteus maximus and vastus lateralis was obtained by plotting the average MEP amplitude against its corresponding percentage of active motor threshold (see Fig. 3). The average and peak slopes of the input–output curve data points were analyzed using linear regression and sigmoid curve fitting, respectively. To calculate the average slope using linear regression, only data points from 100% active motor threshold to the maximum MEP were used (Fig. 3a)^23^. To calculate peak slope, a Boltzman sigmoid function with non-linear least-mean square was fit to data points obtained from all stimulator intensities (Fig. 3b)^15^. The two fitting methods from a sample participant are presented in Fig. 3.

### Statistical analysis

To evaluate between-day test–retest reliability of the peak and average slopes of the input–output curve, as well at the maximum MEP amplitude obtained from vastus lateralis and gluteus maximus, a random-effect intra-class correlation coefficient (ICC) was calculated using PASW statistics 18 (SPSS, Inc.). The ICC_2,1_ and the upper and lower bound of 95% confidence interval were reported for each of the variables. ICC values less than 0.5 were interpreted as poor reliability, ICCs between 0.5 and 0.75 were interpreted as moderate reliability, ICCs between 0.75 and 0.9 were interpreted as good reliability, and ICCs greater than 0.9 were interpreted as excellent reliability^24,25^. The ICC 95% confidence intervals, standard error of measurement and the minimal detectable change were reported for all variables of interest. The standard error of measurement was calculated as (Standard Deviation x √ (1 − ICC)) to determine the random systematic measurement error. The minimal detectable change was calculated as (1.96 × √2 × standard error of measurement).

## Results

**Figure 4:**
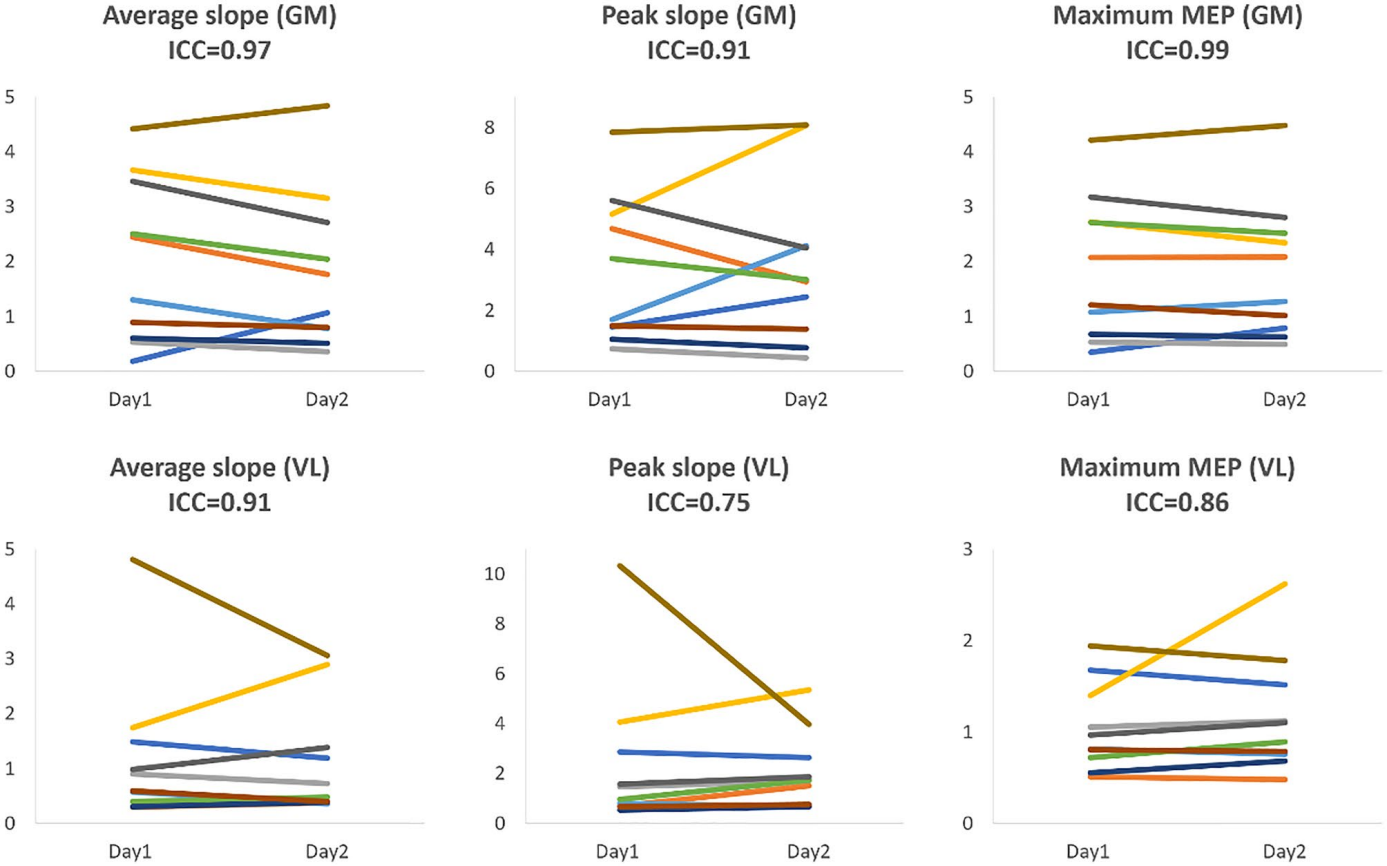
Results of the slopes of the input–output curve from the two fitting methods and maximal MEP from Day 1 and Day 2. Each line in the graphs represents results from Day 1 and Day 2 of the same participant.

All ten participants accomplished all the measurements on both testing days. The active motor thresholds of both gluteus maximus and vastus lateralis for each participant were successfully quantified using our normalized EMG motor thresholding procedure. Group means of the peak-to-peak amplitude of 20% MVIC and active motor threshold of gluteus maximus and vastus lateralis are presented in Table 1. The individual results of the average slope, peak slope and maximum MEP from Day 1 and Day 2 are presented in Fig. 4.

**Table 1:** Descriptive data for active motor threshold (AMT) and the peak to peak amplitude of the 20% maximal voluntary isometric contraction (MVIC) contraction of gluteus maximus (GM) and vastus lateralis (VL).

	Day 1	Day 2
GM 20% MVIC (uV)	336 ± 180	337 ± 178
VL 20% MVIC (uV)	272 ± 53	276 ± 50
GM AMT (%MSO)	32.7 ± 7.69	31.40 ± 8.40
VL AMT (%MSO)	33.70 ± 7.60	33.70 ± 7.18

*MSO* maximal stimulation output.

The reliability coefficients for the TMS measurements as well as the standard error of measurement and minimal detectable change for gluteus maximus and vastus lateralis are presented in Tables 2 and 3, respectively. For gluteus maximus, reliability of average and peak slopes obtained from the input–output curve procedure as well as the maximum MEP achieved excellent reliability (ICCs > 0.9; Table 2). With respect to the vastus lateralis, the reliability of the TMS measures of interest ranged from good to excellent (ICCs ranging from 0.75–0.91; Table 3).

**Table 2:** Reliability results for the TMS measures of interest for gluteus maximus.

	Day 1 (mean ± SD)	Day2 (mean ± SD)	ICC	95% CI	SEM	MDC
Average slope	2.00 ± 1.43	1.80 ± 1.35	0.97	0.87–0.99	0.25	0.70
Peak slope	3.33 ± 2.29	3.53 ± 2.56	0.91	0.63–0.98	0.73	2.03
Maximum MEP (mV)	1.87 ± 1.24	1.84 ± 1.19	0.99	0.96–1.00	0.12	0.34

*SD* standard deviation, *CI* confidence interval, *SEM* standard error of measurement, *MDC* minimal detectable change.

**Table 3:** Reliability results for the TMS measures of interest for vastus lateralis.

	Day 1 (mean ± SD)	Day2 (mean ± SD)	ICC	95% CI	SEM	MDC
Average slope	1.21 ± 1.29	1.12 ± 0.99	0.91	0.63–0.98	0.35	0.96
Peak slope	2.39 ± 2.86	2.08 ± 1.45	0.75	− 0.04–0.94	1.13	3.13
Maximum MEP (mV)	1.04 ± 0.46	1.17 ± 0.61	0.86	0.46–0.96	0.21	0.57

*SD* standard deviation, *CI* confidence interval, *SEM* standard error of measurement, *MDC* minimal detectable change.

## Discussion

With the growing interest in TMS assessment of lower extremity muscles, there is a need to establish methods that are reliable and procedures that allow for valid comparison of outcome measures across muscles. The purpose of the current study was to establish the reliability of a method for measuring corticomotor excitability of proximal lower extremity muscles. By using normalized EMG during the motor thresholding procedure, we sought to minimize 2 potential sources of error that could lead to bias when comparing TMS results across muscles by 1) providing a comparable level of active contraction during thresholding and by 2) establishing a consistent criterion for the identification of MEPs. Importantly, the use of normalized EMG to establish the criterion for motor thresholding as reported in the current study, resulted in the successful and reliable attainment of MEPs from all 10 participants for each muscle on both testing days.

As stated earlier, the assessment of corticomotor excitability for proximal lower extremity muscles is challenging^9,13^. A single-pulse TMS study of gluteus maximus reported that MEP amplitude was only successfully measured in less than one-third of the participants using the traditional fixed voltage value protocol during motor thresholding^9^. It is likely that MEP amplitudes were being attenuated by varying levels of subcutaneous fat overlying gluteus maximus across individuals. Thus, it is likely that MEPs were smaller than the 100uV criteria for an active contraction condition or hidden within the background level of activation.

Data obtained from the input–output curve procedure represents multiple MEPs collected over a range of stimulation intensities and provides a more comprehensive evaluation of corticomotor excitability compared to MEP amplitudes from a single stimulation^15,26^. All TMS measurements related to the input–output curve in the current study demonstrated good to excellent reliability. Although the sigmoid curve fitting is the most common method used to analyze the input–output curve^15,27^, the linear slope method also exhibited excellent reliability. Our range of ICC values were superior to a previous TMS study of the first dorsal interossei that reported poor to excellent reliability (ICCs > ranging from 0.19 to 0.90) for both linear regression and sigmoid curve fitting methods^23^. With respect to lower extremity muscles, our results are consistent with a previous study that reported good reliability of the peak slope obtained from the input–output curve using sigmoid curve fitting for the tibialis anterior (ICC 0.78)^28^.

Our ICC values for maximum MEP are somewhat better than previous reliability studies in which the gluteus maximus and vastus lateralis were evaluated. For example, the ICCs for test–retest reliability for MEP amplitude at a single stimulation intensity has been reported to range from 0.76–0.83 for gluteus maximus^12^ and 0.82–0.97 for vastus lateralis^29^. Our relatively low values for the standard error of measurement and minimal detectable change for maximum MEPs of vastus lateralis and gluteus maximus indicate that such measures would be sensitive enough to detect real change owing to an intervention or to identify differences between muscles.

Previous studies have suggested that 20–30 stimulations are required to obtain the most reliable corticomotor excitability using MEP amplitudes as the outcome variable^30,31^. However our findings demonstrate that 10 stimulations at each of the stimulator intensities was sufficient to yield good to excellent reliability using the input–output procedure. It could be argued that the good to excellent levels of reliability reported in the current study may be attributed to the use of normalized EMG to provide a consistent level of active contraction during thresholding and to establish a standardized criterion for the determination of MEPs. This is important as previous research has reported that MEP amplitude of gluteus maximus can be biased with greater muscle activation levels^32^. With respect to vastus lateralis, Temesi et al. reported the peak input–output curve slope during 50% MVIC was significantly steeper than the peak slope obtained during 10% MVIC^33^. It is clear that MEP amplitudes are influenced by the contraction level and would be a source of unwanted variability. Controlling for such variability would appear to be most important in evaluating maximum MEP or the MEP at a single stimulator intensity.

For participants in which significant subcutaneous fat is present, use of the 100uV criteria would require that the stimulator intensity be continually increased to achieve a MEP. Using normalized EMG, the MEP amplitude and the criteria are expressed as a percentage of MVIC which would require a lower stimulator intensity to achieve a MEP. Therefore, the motor thresholding would be more tolerable for participants as the stimulation intensity would be minimized as well as the number of required stimulations. On the other end of the spectrum, the background EMG from a pre-stimulus contraction can be larger than 100 uV criteria and therefore MEPs may be embedded in the background EMG signal and difficult to detect. Applying this methodology is particularly important when more than one muscle (ie. gluteus maximus and vastus lateralis) is examined and compared in the same study using an active contraction condition during the TMS procedure.

There are several limitations of our study that should be considered when interpreting the results. First, we only recruited young, healthy participants. As such, our findings may not be generalizable to various patient populations. Second, the TMS procedure described above was performed with participants performed an isometric contraction in a supine position. Our findings may not apply to TMS procedures performed in standing or during active movement (i.e. walking). Third, we only evaluated the reliability of TMS outcome measures related to the input–output curve. Whether or not other measures would exhibit the high levels of reliability reported here remains to be determined (i.e. MEP latency, cortical silent period). Fourth, this study only evaluated a relatively small sample (10 participants). Lastly, the use of normalized EMG for the purposes of this study assumed that all participants provided a maximum effort during the MVIC.

## Conclusion

A reliable method to measure corticomotor excitability of proximal lower extremity muscles has been described. Specifically, we used normalized EMG to (1) provide a consistent target contraction level during the motor thresholding procedure and (2) establish a consistent criterion for the identification of a MEP (as opposed to a fixed voltage). Better standardization of TMS procedures is necessary to facilitate comparison of outcome measures between lower extremity muscles and individuals. Thus, this method affords the ability to determine neuroplastic changes in the acquisition of lower extremity functional tasks.

## Data Availability

The datasets generated during and/or analysed during the current study are available from the corresponding author on reasonable request.
